# Alzheimer’s disease candidate gene PION is differentially expressed in human prefrontal cortex

**DOI:** 10.1186/1750-1326-7-S1-S25

**Published:** 2012-02-07

**Authors:** Min Zhu, David Saffen

**Affiliations:** 1 Institutes of Brain Science, State Key Laboratory for Medical Neurobiology, School of Life Science, Fudan University, China

## Background

*PION* (Pigeon homologue protein), located on chromosome 7q11.23, encodes gSAP (gamma-secretase activating protein), which is cleaved to form a 16 kDa C-terminal fragment (gSAP-16K) that interacts with gamma-secretase to facilitate the cleavage of amyloid precursor protein-beta-C-terminal fragment (APP-beta-CTF), releasing the neurotoxic peptide A-beta [He G, et al, *Nature* 2010]. The study by He G, *el al*. also demonstrated that partial siRNA-mediated inhibition of gSAP expression in N2a cells reduces A-beta production, suggesting that gSAP-16K modulates APP-beta-CTF cleavage in a dose-dependent manner. Taken together, these observations suggest that levels of *PION* gene expression in human brain may also affect levels of A-beta production and thereby influence the risk of developing Alzheimer’s disease. The goal of the present study is to quantify variation of *PION* mRNA expression in human prefrontal cortex and identify haplotypes and/or combinations of genotypes that predict high- or low-levels of mRNA expression.

## Methods

Sixty-four independent frozen sections of prefrontal cortex (Han Chinese autopsy samples; Brodmann area 46) were obtained from the China Brain Bank Center (Wuhan, China). Genomic DNA and total RNA were isolated using standard techniques. A common SNP, rs2037753 (heterozygosity = 0.422), located within exon 16 of *PION* mRNA was chosen as a molecular marker to distinguish mRNAs derived from each autosomal allele. SNaPShot^©^-based AEI assays were carried out as previously described [Lim JE, *Molecular Psychiatry*, 2007]. Analysis of population distributions of log_2_AEI ratios was carried out using a mathematical model developed in-house. Levels of *PION* mRNA relative to mRNA encoding the house-keeping gene *GAPDH* were quantified by real-time PCR (calculated as ΔCt). Genomewide genotyping of all the above samples was carried out using HumanOmni1-Qad arras (Illumina) arrays. SNPs with genotypes that correlate with relative expression of *PION* mRNA were identified by linear regression analysis for SNPs within a 205,650 base pair region of chromosome 7 centered on *PION*.

## Results

We observed robust allele-specific expression of *PION* mRNA in approximately 24 out of 34 samples heterozygous for the marker SNP. Mathematical modeling of the log_2_AEI population distributions predicted that *PION* mRNA expression is controlled by three *cis*-acting regulatory elements, one of which is in partial linkage disequilibrium with the marker SNP. Scanning SNPs in the region of the *PION* gene for correlations with mRNA expression revealed a single SNP, rs10271991 (located in intron 22), that is highly correlated with mRNA expression (r2 = 23.5; P =0.0001). Comparison between a model that includes rs10271991 (Major allele frequency = 0.51; D’ with marker SNP = 0.8) and experimentally determined log_2_AEI distributions yielded excellent agreement (r^2^ = 0.96). According to this model, the remaining two variants that control expression of PION mRNA are both predicted to have major allele frequencies of approximately 0.8 and to be unlinked to the marker SNP (i.e., D’ = 0).

## Conclusions

We have used real-time PCR-based measurements of relative mRNA expression in human prefrontal cortex and genotyping to identify a SNP within the *PION* gene that correlates with mRNA expression. Our modeling of population distributions of log_2_AEI ratios predicts that *PION* mRNA expression is controlled by a variant linked to this SNP plus two additional variants that remain to be identified.

